# Global Gene Expression Profiling of a Population Exposed to a Range of Benzene Levels

**DOI:** 10.1289/ehp.1002546

**Published:** 2010-12-13

**Authors:** Cliona M. McHale, Luoping Zhang, Qing Lan, Roel Vermeulen, Guilan Li, Alan E. Hubbard, Kristin E. Porter, Reuben Thomas, Christopher J. Portier, Min Shen, Stephen M. Rappaport, Songnian Yin, Martyn T. Smith, Nathaniel Rothman

**Affiliations:** 1 School of Public Health, University of California–Berkeley, Berkeley, California, USA; 2 Occupational and Environmental Epidemiology Branch, Division of Cancer Epidemiology and Genetics, National Cancer Institute, National Institutes of Health, Department of Health and Human Services, Bethesda, Maryland, USA; 3 Institute of Risk Assessment Sciences, Utrecht University, Utrecht, the Netherlands; 4 Institute of Occupational Health and Poison Control, Chinese Center for Disease Control and Prevention, Beijing, China; 5 Environmental Systems Biology Group, Laboratory of Molecular Toxicology, National Institute of Environmental Health Sciences, National Institutes of Health, Department of Health and Human Services, Research Triangle Park, North Carolina, USA

**Keywords:** benzene, biomarker, human, microarray, transcriptomics

## Abstract

**Background:**

Benzene, an established cause of acute myeloid leukemia (AML), may also cause one or more lymphoid malignancies in humans. Previously, we identified genes and pathways associated with exposure to high (> 10 ppm) levels of benzene through transcriptomic analyses of blood cells from a small number of occupationally exposed workers.

**Objectives:**

The goals of this study were to identify potential biomarkers of benzene exposure and/or early effects and to elucidate mechanisms relevant to risk of hematotoxicity, leukemia, and lymphoid malignancy in occupationally exposed individuals, many of whom were exposed to benzene levels < 1 ppm, the current U.S. occupational standard.

**Methods:**

We analyzed global gene expression in the peripheral blood mononuclear cells of 125 workers exposed to benzene levels ranging from < 1 ppm to > 10 ppm. Study design and analysis with a mixed-effects model minimized potential confounding and experimental variability.

**Results:**

We observed highly significant widespread perturbation of gene expression at all exposure levels. The AML pathway was among the pathways most significantly associated with benzene exposure. Immune response pathways were associated with most exposure levels, potentially providing biological plausibility for an association between lymphoma and benzene exposure. We identified a 16-gene expression signature associated with all levels of benzene exposure.

**Conclusions:**

Our findings suggest that chronic benzene exposure, even at levels below the current U.S. occupational standard, perturbs many genes, biological processes, and pathways. These findings expand our understanding of the mechanisms by which benzene may induce hematotoxicity, leukemia, and lymphoma and reveal relevant potential biomarkers associated with a range of exposures.

Benzene is an established cause of acute myeloid leukemia (AML) and myelodysplastic syndromes, and is a probable cause of lymphocytic malignancies (Baan et al. 2009; Vlaanderen et al. 2010), including non-Hodgkin lymphoma (NHL) in humans, as recently reviewed by Smith (2010). Benzene is also hematotoxic, even at relatively low levels of exposure (Lan et al. 2004). Possible mechanisms underlying these pathologies include the generation of free radicals leading to oxidative stress, immune system dysfunction, and decreased immune surveillance (Smith 2010). Studies of global gene expression in the bone marrow of very highly exposed mice have revealed additional potential mechanisms of benzene toxicity (Faiola et al. 2004; Yoon et al. 2003), but their relevance to risk in occupationally exposed individuals is uncertain. Toxicogenomic studies of exposed human populations are an important alternative approach to the human health risk assessment of environmental exposures. Such studies that have examined environmental exposures have identified potential biomarkers of early effects and revealed potential mechanisms underlying associated diseases (McHale et al. 2010). However, these studies have been of limited size, have mainly addressed high levels of exposure, and have often lacked precise, individual estimates of exposure. Further, such studies are limited by confounding effects and laboratory variation, especially at low doses.

We previously compared global gene expression in the peripheral blood mononuclear cell (PBMC) fractions of six to eight pairs of unexposed controls and workers exposed to high levels of benzene (> 10 ppm) and identified potential biomarkers of exposure and mechanisms of toxicity (Forrest et al. 2005; McHale et al. 2009). We chose PBMCs because they are widely used in human toxicogenomic studies. As an extension of these earlier studies, here we sought to identify potential gene expression biomarkers of exposure and early effects, as well as mechanisms of toxicity, in 125 individuals occupationally exposed to a range of benzene levels, including < 1 ppm, the current U.S. occupational standard (Occupational Safety and Health Administration 1987). In the cross-sectional molecular epidemiological study population, which includes the 125 individuals analyzed here, we previously found that white blood cell counts were decreased in workers exposed to < 1 ppm benzene compared with controls and that a highly significant dose–response relationship was present (Lan et al. 2004), with no apparent threshold within the occupational exposure range (0.2–75 ppm benzene) (Lan et al. 2006). We employed a rigorous study design that included randomization of samples across experimental variables, incorporation of precise individual measurements of exposure, and analysis with a mixed-effects model, with the aim of removing sources of biological and experimental variability (nuisance variability).

## Materials and Methods

### Study subjects and exposure assessment

All subjects were from a molecular epidemiology study of occupational exposure to benzene that comprised 250 benzene-exposed shoe manufacturing workers and 140 unexposed age- and sex-matched controls who worked in three clothes-manufacturing factories in the same region near Tianjin, China (Lan et al. 2004; Vermeulen et al. 2004). This study complied with all applicable requirements of U.S. and Chinese regulations, including institutional review board approval. Participation was voluntary, and written informed consent was obtained.

Exposure assessment to benzene was performed as described previously (Vermeulen et al. 2004). For this study, we categorized exposure groups using mean individual air benzene measurements obtained during the 3 months preceding phlebotomy. A subgroup of subjects was selected from each benzene exposure category as follows: 13 workers with very high exposure (> 10 ppm), 11 workers with high exposure (5–10 ppm), 30 workers with low exposure (< 1 ppm; average < 1 ppm), and 29 workers with very low exposure (<< 1 ppm; average < 1 ppm, with most individual measurements < 1 ppm) (Table 1). We previously reported that urinary benzene and mean individual air levels of benzene were strongly correlated (Spearman *r* = 0.88, *p* < 0.0001) in the epidemiological study population (Lan et al. 2004). Among the individuals with occupational exposure to benzene in the present study for which urinary benzene levels were available (*n* = 82), a similar correlation was noted (Spearman *r* = 0.76, *p* < 0.0001). A group of 42 unexposed controls were frequency matched to the exposed subjects on the basis of age and sex. Mean age (± SD) was 29.5 ± 8.7 years for the 83 exposed workers and 29.5 ± 8.2 years for the controls.

Biological sample collection was described previously (Forrest et al. 2005; Vermeulen et al. 2004). We transferred field-stabilized samples on dry ice. We isolated RNAs using the *mir*Vana miRNA (microRNA) isolation kit (Applied Biosystems, Austin, TX, USA), stored them in aliquots at −80°C, and thawed them immediately before microarray analysis. All RNA samples analyzed had absorbance ratios for A_260_:A_280_ and A_260_:A_230_ between 1.7 and 2.1, and we confirmed integrity by the presence of sharp 28S and 18S rRNA bands and a ratio of 28S:18S intensity of approximately 2:1 after denaturing gel electrophoresis.

### Microarray study design and analysis

We randomized samples, and thus exposure groups, across labeling and hybridization reactions and across chips as uniformly as possible [see Supplemental Material, Table 1 (doi:10.1289/ehp.1002546)]. Technical replicates (*n* = 19), randomly chosen from among the 125 study subject samples, were included in the study to assess variability in the labeling, hybridization, and chip steps of the microarray procedure. We labeled samples (200 ng) in batches of 24 using the Illumina RNA Amplification kit (Ambion, Austin, TX, USA) and hybridized them to Illumina HumanRef-8 V2 BeadChips in batches of 32 (four chips) following the manufacturer’s protocol. All sample processing was performed in a blinded manner.

### Data analysis

We conducted variance components analysis using a linear mixed model (Laird and Ware 1982) to assess the proportion of total variation due to variation between subjects, hybridizations, labels, and chips, both before and after normalization [quantile normalization in the affy package (Gautier et al. 2004) in R (R Development Core Team 2010)]. For each probe, we estimated the association between exposure level and expression level using a mixed-effects model with random intercepts that accounted for clustering by subject, hybridization, and label. The fixed effects in our model, in addition to benzene exposure level, included sex (1 = male, 0 = female), current smoking status (1 = yes, 0 = no), and age (in years, linear term) as potential confounders of associations between gene expression and benzene exposure. We fitted the mixed-effects model in R with the lmer function in the lme4 package (Bates and Maechler 2010). We identified differentially expressed probes as those with a statistically significant log-fold change (based on likelihood ratio tests). We computed *p*-values adjusted for multiple testing by controlling the false discovery rate (FDR) with the Benjamini-Hochberg procedure (Benjamini and Hochberg 1995), using the multtest package in R. These values are FDR-adjusted *p*-values and were considered significant if they were ≤ 0.05, the traditional experiment-wise type I error rate. The raw data discussed here have been deposited in the National Center for Biotechnology Information (NCBI) Gene Expression Omnibus (GEO) (Edgar et al. 2002) and are accessible through the GEO database (accession number GSE21862; NCBI 2002).

### Pathway analysis

We imported microarray probe IDs into Pathway Studio software (Ariadne Genomics, Rockville, MD, USA), and queried the ResNet 7.0 database (Ariadne Genomics) for interactions among genes and gene products derived from the current literature (Nikitin et al. 2003). We also used a method known as “structurally enhanced pathway enrichment analysis” (SEPEA_NT3) (Thomas et al. 2009), which incorporates the associated network information of KEGG (Kyoto Encyclopedia of Genes and Genomes) biochemical pathways (Kanehisa and Goto 2000; Kyoto Encyclopedia of Genes and Genomes 2000). KEGG pathways are manually drawn pathway maps representing current knowledge on the molecular interaction and reaction networks involved in cellular processes such as metabolism and the cell cycle.

### Gene Ontology (GO) analysis

The GO project (The Gene Ontology Consortium 2000) provides an ontology of defined terms representing gene product properties in the domains, cellular components, molecular functions, and biological processes. GO has a hierarchical structure that forms a directed acyclic graph in which each term has defined relationships to one or more other terms in the same domain, which can be described as parent–child relationships. Every GO term is represented by a node in this graph, and the nodes are annotated with a set of genes. We used TopGO (topology-based GO scoring; Bioconductor 2010) to calculate the significance of biological terms from gene expression data taking the GO structure into account (Alexa et al. 2006). We used the “elim” algorithm, which differs from standard GO analyses in that it eliminates genes from parent nodes that are members of “significant” child nodes. The elim score is the *p*-value returned by Fisher’s exact test, and a node is marked as significant if the *p*-value is smaller than a previously defined threshold (Alexa et al. 2006). Typically this threshold is set to be 0.01 divided by the number of nodes in the GO graph with at least one annotated gene. This corresponds to a Bonferroni adjustment of the *p*-values. The most highly significant nodes thus derived are denoted as key nodes.

Both TopGO and SEPEA_NT3 have limitations (Barry et al. 2005; Nettleton et al. 2008). They assume independence between expressions of the genes, violation of which can lead to greater false positives than allowed by the nominal threshold set. These methods were chosen over more computationally intensive permutation-based subject sampling approaches.

### Hierarchical clustering

We performed simple supervised clustering based on complete linkage (Murtagh 1985) in order to make heat maps [hierarchical agglomerative clustering with complete linkage; implemented in the hclust function in R (R Development Core Team 2010), called by the heatmap.2 function available with the gplots library in Bioconductor (Gentleman et al. 2004)]. Input data consisted of the four columns of log_2_-adjusted ratios (the coefficients from the linear mixed-effects models adjusted for both random and fixed effects). This provides clusters driven by average responses within dose groups rather than by potential confounding within groups.

## Results

### Application of a mixed-effects model to analyze gene expression

**Figure 1:**
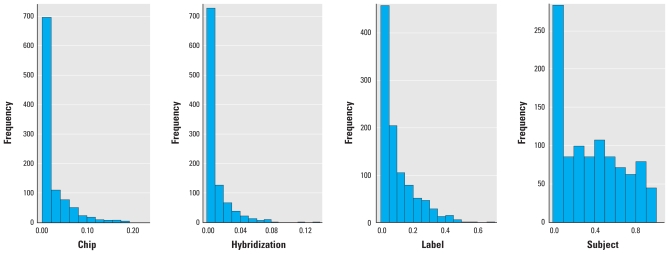
Distribution of the intraclass correlation coefficients (the proportion of variability estimated to come from each source on a probe-by-probe basis) calculated by variance components analysis based on a mixed-effects model allowing assessment of independent contributions of variability from chip, hybridization, label, and biological (subject), as well as residual variability.

We applied a mixed model (variance components analysis) to assess the proportion of total variation due to variation among subjects, hybridizations, labels, and chips, among the randomly selected within-subject replicates (*n* = 19). Plotting the distribution of the contribution of variance across all probes after normalization revealed that the greatest source of variation was between subjects and was therefore consistent with biological causes (Figure 1). We also found substantial variation between labeling reactions. Therefore, for each probe, we estimated the association between exposure level and expression level using a mixed-effects model with (crossed) random intercepts that account for clustering by subject and by label (Laird and Ware 1982). Because the study design included randomization of samples—and thus exposures—across labeling reactions, an inferential procedure was necessary that allowed the existence of nonnested sources of correlation (labeling and subject). Thus, we used mixed models with so-called crossed random effects (Fitzmaurice et al. 2004), with the goal of providing more trustworthy inference than procedures that would have ignored, for instance, the variability caused by the labeling. (Many microarray studies are not designed to partition out the sources of variability and thus, if such sources are important, could provide misleading inference. In addition, it is often assumed that normalization will eliminate these sources of variability, but this assumption cannot be verified unless the study design allows for partitioning of the variance.) In the model, we also adjusted, as simple fixed effects, for biological variation in expression associated with differences in sex, age, and smoking status.

### Effects of benzene exposure on gene expression, biological processes, and pathways

Analysis of the overall effect of benzene across the four exposure categories (very high, high, low, and very low) relative to unexposed controls (*n* = 42) revealed significantly altered expression (FDR-adjusted *p*-values ≤ 0.05) of 3,007 probes representing 2,846 genes [see Supplemental Material, Table 2 (doi:10.1289/ehp.1002546)]. Immune response (*p* = 3.78E-07) was the most significant key node among the GO processes associated with exposure (see Supplemental Material, Table 3), as determined by TopGO analysis. Pathway analysis by SEPEA_NT3 (Thomas et al. 2009) revealed highly significant (*p* < 0.001) impacts on the Toll-like receptor signaling pathway, oxidative phosphorylation, B-cell receptor signaling pathway, apoptosis, AML, and T-cell receptor signaling (see Supplemental Material, Table 4).

Large numbers of genes were significantly differentially expressed (FDR-adjusted *p*-values ≤ 0.05) in samples from each of the four exposure categories relative to controls [see Supplemental Material, Figure 1 and Tables 5–8 (doi:10.1289/ehp.1002546)]. We identified several GO processes implicated in the overall analysis as key nodes across three to four dose categories, including immune response, apoptosis, and ATP synthesis– coupled proton transport [Table 2; for complete data, see Supplemental Material, Table 9).

Similarly, multiple pathways found to be highly significant in the overall analysis (*p* ≤ 0.005), including Toll-like receptor signaling, oxidative phosphorylation, B-cell receptor signaling, apoptosis, AML, and T-cell receptor signaling, were enriched among the differentially expressed genes associated with three (including the very low dose category) or four exposure categories [Table 3; for complete data, see Supplemental Material, Table 10 (doi:10.1289/ehp.1002546)].

**Figure 2:**
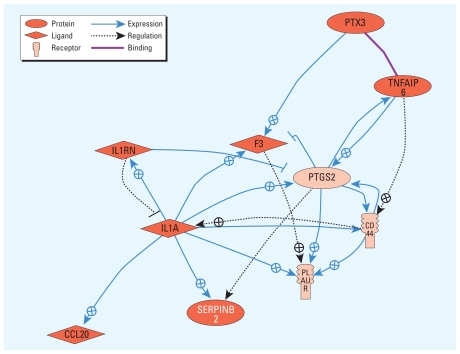
Network interactions among biomarkers of benzene exposure associated with all exposure levels, illustrating a high degree of interrelatedness based on the literature, with central roles for *IL1A* and *PTGS2*. Pathway Studio software identified interactions among 10 of the 16 potential biomarkers of benzene exposure. The interactions are mainly expression, with some regulation (regulator changes the activity of the target) and one binding interaction. Red indicates up-regulation.

Twelve genes were up-regulated ≥ 1.5-fold at all four doses relative to unexposed controls, including five genes [*PTX3* (pentraxin-related gene), *CD44* (CD44 antigen), *PTGS2* (prostaglandin-endoperoxide synthase 2), *IL1A* (interleukin 1, alpha), and *SERPINB2* (serpin peptidase inhibitor, clade B, member 2) with FDR-adjusted *p*-values ≤ 0.005. An additional four genes were up-regulated > 1.5-fold at the top three doses, and > 1.3-fold at the lowest dose (Table 4). Expression of each of the 16 signature genes across the five exposure categories shows a distinct pattern, with the highest expression in the < 1-ppm (low) exposure group [see Supplemental Material, Figure 2 (doi:10.1289/ehp.1002546)]. The 16 genes are involved in immune response, inflammatory response, cell adhesion, cell–matrix adhesion, and blood coagulation (see Supplemental Material, Table 11). Ten of the 16 genes (or their products), 7 of which are involved in inflammatory response (*p* = 1.4E-12), form a network (Figure 2) with central roles for *IL1A* and *PTGS2.*

### Dose-specific effects

**Figure 3:**
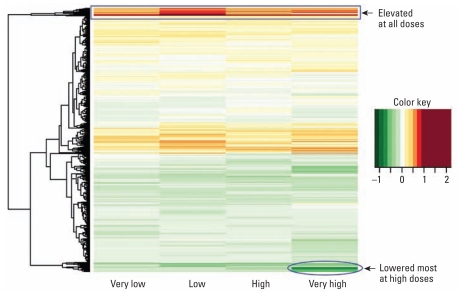
Dose-dependent effects on gene expression. A heat map illustrates simple hierarchical clustering of the differentially expressed 3,007 probes (FDR-adjusted *p*-value < 0.05) based on the mixed model described in “Materials and Methods.” The clustering was done on the four log_2_ expression ratios (derived as coefficients returned from the mixed model) all relative to controls. The color key relates to the log_2_ ratios observed. Clustering of genes was based on complete linkage (for algorithmic details of algorithms used, see Murtagh 1985), as implemented in the hclust function in R, called by the heatmap.2 function available with the gplots library in Bioconductor (Gentleman et al. 2004). Note that the clustering is based on Euclidean distance.

We used supervised hierarchical clustering to generate a heat map to allow visualization of patterns of gene expression across exposure categories. One group of genes (~ 100) exhibited reduced expression (ratios < 1) with increasing dose relative to controls, whereas a second group (~ 100) appeared to be elevated at all doses but more so at low-dose exposure (Figure 3).

We also observed dose-dependent effects on biological processes and pathways. For example, nucleosome assembly [see Supplemental Material, Table 9 (doi:10.1289/ehp.1002546)] and the ATP-binding cassette (ABC) transporter pathway (see Supplemental Material, Table 10) appeared to be deregulated only at the very high exposure level. Among 78 genes that were highly significantly (FDR *p*-value ≤ 0.05) associated with a ≥ 1.5-fold increase in expression in the very high exposure group, and not significantly altered at any of the other exposure categories relative to controls, a network involving 19 genes (or their products) was apparent, in which v-src sarcoma viral oncogene homolog (*SRC*) and matrix metallopeptidase 9 (*MMP9*) play central roles (see Supplemental Material, Figure 3). Among 29 genes significantly altered only at low-dose benzene exposure, we identified a network of 15 genes involved in immune response (*p* = 4E-12), with central roles for interferon gamma (*IFNG*) and tumor necrosis factor (*TNF*) (see Supplemental Material, Figure 4). Together, these data suggest that benzene induces dose-dependent effects, with the caveat that differences in power among the different exposure categories may have influenced the resulting significant gene lists.

## Discussion

Technical variation is often ignored in human toxicogenomic studies, leading to potential bias in differential expression arising from correlation with technical variation. In the present study, we applied a rigorous study design to assess sources of both potential confounding and experimental variability (nuisance variation) and analyzed the data using statistical techniques that incorporate nonnested sources of variation (i.e., those not eliminated by normalization) and that return estimates of least variability with accurate inference (linear mixed-effects models). This approach increased the power to detect associations between benzene exposure and gene expression, even at low-dose exposure levels.

More genes remained significantly up- or down-regulated compared with controls after multiple test correction in the present study than in an earlier study examining samples from eight pairs of exposed workers and unexposed controls on the Illumina platform (McHale et al. 2009), likely because of the increased number of individuals and the rigorous approach to study design. Nonetheless, we identified 247 genes in both study populations using the Illumina platform. Of 488 significant genes cross-validated on both Illumina and Affymetrix platforms (McHale et al. 2009), 147 genes were significant in the present study. *ZNF331* (zinc finger protein 331), significant after multiple test correction in individuals occupationally exposed to benzene at levels > 10 ppm compared with controls in two earlier studies (Forrest et al. 2005; McHale et al. 2009), was significantly up-regulated at both < 1 ppm and > 10 ppm in the present study.

The finding that genes in the AML pathway were strongly associated with multiple exposure levels of benzene provides support for our approach because epidemiological studies have established that benzene causes AML (Baan et al. 2009; Smith 2010). However, such disease associations must be treated cautiously because the KEGG pathway information, on which the pathway analyses were based, is limited for AML, and a KEGG pathway for NHL has not been defined. Information about altered molecular and cellular processes can provide biological plausibility for probable disease associations. Immune response, previously found to be associated with > 10 ppm benzene exposure in our earlier transcriptomic study of eight high-exposed control pairs (McHale et al. 2009), was one of the major processes significantly altered across multiple exposure levels in the present study, involving both innate (Toll-like receptor signaling) and adaptive (B-cell receptor signaling and T-cell receptor signaling pathway) responses. Additionally, we found central roles for the proinflammatory cytokines *IFNG* and *TNF* among genes uniquely altered at low-dose exposure in the present study. A single nucleotide polymorphism in *TNF*-α was previously associated with susceptibility to bone marrow dysplasia in chronic benzene poisoning (Lv et al. 2007). Further, genetic variation in *TNF* (Rothman et al. 2006), Toll-like receptor genes (Purdue et al. 2009), and *IFNG* (Colt et al. 2009) has previously been associated with NHL risk. Deregulation of pathways involving these genes through sustained alterations in expression provides biological plausibility for the association of benzene with lymphoid neoplasms.

Findings from the present study are consistent with previous reports of adverse effects of benzene on oxidative stress (Kolachana et al. 1993) and mitochondria (Inayat-Hussain and Ross 2005). Here, we found highly significant associations with ATP synthesis–coupled proton transport and oxidative phosphorylation at all levels of benzene exposure relative to unexposed controls. Expression of superoxide dismutase (*SOD*), a mitochondrial defense against reactive oxygen species, was up-regulated in the present study by 50–100% relative to controls. *HMOX1* [heme oxygenase (decycling) 1], an antioxidant and suppressor of TNF-α signaling (Lee et al. 2009), was down-regulated in the low-dose benzene exposure group. Increased mitochondrial membrane permeability potential induced by benzene metabolites (Inayat-Hussain and Ross 2005) can lead to the initiation of apoptosis. Indeed, apoptosis was associated with all benzene doses in the present study, consistent with our earlier observation of an association with high-dose benzene exposure (> 10 ppm) (McHale et al. 2009).

Previously, we found that chromatin assembly was significantly altered after high-dose benzene exposure (McHale et al. 2009). The finding that nucleosome assembly (a GO category nested within chromatin assembly) was overrepresented in the highest exposure category in the present study confirms and clarifies this potential mechanism of benzene-associated leukemia.

Although significant involvement of the p53 response pathway was previously found in mice exposed to very high levels of benzene (Faiola et al. 2004; Yoon et al. 2003), we did not find such involvement in the present study or in our earlier studies, and the immune and inflammatory effects we found here in humans were not recapitulated in the mouse microarray studies (Faiola et al. 2004; Yoon et al. 2003). These differences suggest that human toxicogenomic studies may be more relevant than animal studies, although differences in exposure levels, tissues examined, and uncontrolled confounding in the human study could also be contributing factors.

Our findings suggest two novel hypotheses regarding benzene toxicity. Glycosylphosphatidylinositol (GPI)-anchor biosynthesis was associated with all doses of benzene exposure in the present study. The GPI anchor is a C-terminal posttranslational modification that anchors the modified protein in the outer leaflet of the cell membrane and putatively plays roles in lipid raft partitioning, signal transduction, and cellular communication (Paulick and Bertozzi 2008). Because epigenetic silencing of genes involved in GPI-anchor biosynthesis may be important in human disease, including lymphomas (Hu et al. 2009), further investigation of its role in benzene-associated disease is warranted.

ABC transporters were associated highly significantly with only the highest (> 10 ppm) benzene dose. In addition to their capacity to extrude cytotoxic drugs, ABC transporters are known to play important roles in the development, differentiation, and maturation of immune cells and are involved in migration of immune effector cells to sites of inflammation (van de Ven et al. 2009).

Our findings also suggest a potential gene expression signature of benzene exposure. In particular, *IL1A* and *PTGS2* played central roles in the interaction network characterizing the gene expression signature associated with benzene in this study. Both molecules are produced by activated macrophages and other cells in inflammatory responses. A single nucleotide polymorphism that increases *IL1A* mRNA expression has been inversely associated with granulocyte count in benzene- exposed individuals (Lan et al. 2005). Overexpression of *PTGS2*, which occurs frequently in premalignant and malignant neoplasms, including hematological malignancies (Bernard et al. 2008), together with overexpression of the prostaglandin cascade, leads to carcinogenesis through a progressive series of highly specific cellular and molecular changes (Harris 2009).

The expression pattern of the signature genes suggests a nonlinear response to benzene. Other biomarkers evaluated in populations exposed to benzene have shown similar patterns, including hematotoxicity (Lan et al. 2004), benzene metabolism (Kim et al. 2006), and the generation of protein adducts (Rappaport et al. 2002, 2005). Further characterization of the expression levels of these genes across a range of benzene exposures in a larger, independent study is necessary to determine the applicability of the signature genes as biomarkers of early effects and to explore more formally the shape of the dose–response curve.

## Conclusion

We have identified gene expression biomarkers of early effects across a range of benzene exposures. Our findings support previously reported mechanisms relevant to adverse effects of benzene and suggest potential novel mechanisms for benzene toxicity. Future work should include validation of the potential biomarkers and determining whether the gene expression changes are effected through epigenetic processes such as DNA methylation (Bollati et al. 2007) and miRNA expression.

## Figures and Tables

**Table 1:** Characteristics of study subjects.

Benzene exposure category (ppm)	Subjects (*n*)	Air benzene (ppm)^a^	WBC count (per μL blood)	Age (years)	Sex [*n* (%)]		Currently smoking [*n* (%)]	
					Male	Female	Yes	No
Control (—)	42	< 0.04^b^	6454.8 ± 1746.5	29.5 ± 8.2	17 (33)	25 (34)	9 (35)	33 (33)
Very low (<< 1)^c^	29	0.3 ± 0.9	5524.1 ± 1369.2	30.3 ± 9.2	8 (16)	21 (28)	6 (23)	23 (23)
Low (< 1)^d^	30	0.8 ± 0.8	5510.0 ± 1170.7	27.9 ± 7.2	19 (37)	11 (15)	5 (19)	25 (25)
High (5–10)	11	7.2 ± 1.3	5418.2 ± 1376.8	29.7 ± 9.1	1 (2)	10 (14)	1 (4)	10 (10)
Very high (> 10)	13	24.7 ± 15.7	5176.9 ± 1326.8	30.9 ± 10.5	6 (12)	7 (9)	5 (19)	8 (8)

WBC, white blood cell. Values for air benzene, WBC count, and age are mean ± SD.

a Air benzene level in the 3 months preceding phlebotomy.

b The limit of detection for benzene was 0.04 ppm (Lan et al. 2004).

c The average level of benzene was < 1 ppm and dosimetry levels were < 1 ppm at most measurements in the 3 months preceding phlebotomy and at all measurements in the prior month.

d The average level of benzene was < 1 ppm (in the 3 months preceding phlebotomy) but dosimetry levels were not always < 1 ppm in the previous 3 months.

**Table 2:** Summary of GO categories overrepresented at each benzene exposure category.

GO ID^a^	GO term	Total no. of genes^b^	Very low (*n* = 29)		Low (*n* = 30)		High (*n* = 11)		Very high (*n* = 13)	
			No. genes	*p*-Value^c^	No. genes	*p*-Value^c^	No. genes	*p*-Value^c^	No. genes	*p*-Value^c^
GO:0006412	translation	456	64	2.0E-06	93	1.2E-03				
GO:0006512	ubiquitin cycle	480	48	7.5E-04	98	1.6E-05				
GO:0006917	induction of apoptosis	216	27	4.1E-04	49	1.6E-04	19	1.5E-03^d^		
GO:0006955	immune response	653	58	3.7E-03^d^	124	4.6E-05	54	4.9E-06	97	1.1E-04
GO:0015986	ATP synthesis coupled proton transport	40	11	2.2E-05	14	5.0E-04			11	1.8E-03
GO:0006915	apoptosis	804	80	5.6E-03	158	9.2E-04			107	2.7E-03
GO:0030301	cholesterol transport	8	5	4.4E-05	4	1.5E-02^d^			4	5.5E-03^d^
GO:0006954	inflammatory response	318			60	4.6E-03^d^	34	2.8E-05		

a GO categories that are significant at ≥ 2 doses.

b Number of annotated genes included on the chip.

c *p*-Values were determined using the elim method in TopGO, which computes the statistical significance of a parent node dependent on the significance of its children by Fisher’s exact test; nodes are significant if the *p*-value is smaller than a previously defined threshold (Alexa et al. 2006), 0.01 divided by the number of nodes in the GO graph with at least one annotated gene.

d Significantly enriched term in classic analysis (which does not take GO hierarchy into account) but not in elim analysis in TopGO. Complete GO data are available in Supplemental Material, Table 9 (doi:10.1289/ehp.1002546).

**Table 3:** *p*-Values for pathways altered at each benzene exposure category.

	Benzene exposure category			
Pathway name^a^	Very low (*n* = 29)	Low (*n* = 30)	High (*n* = 11)	Very high (*n* = 13)
Chronic myeloid leukemia	0.034	0.033		
Pancreatic cancer	0.023	0.007		
Oxidative phosphorylation^b^	< 0.001	0.003	0.001	
Small-cell lung cancer^b^	0.004	0.002	0.027	
B-cell receptor signaling pathway^b^	0.008	0.003	0.004	
Insulin signaling pathway	0.015	0.035	0.052	
Adipocytokine signaling pathway	0.034	0.002	0.019	
Circadian rhythm—mammal	0.04	0.045	0.004	
RNA polymerase	< 0.001		0.048	
Toll-like receptor signaling pathway^b^	< 0.001	0.002	0.001	0.004
Epithelial cell signaling in *Helicobacter pylori* infection^b^	< 0.001	0.003	0.006	0.011
GPI-anchor biosynthesis^b^	< 0.001	0.041	< 0.001	0.007
T-cell receptor signaling pathway^b^	0.005	0.002	0.005	0.018
Apoptosis^b^	0.007	0.002	0.007	0.013
Cytokine–cytokine receptor interaction^b^	0.036	0.011	0.030	0.004
AML^b^	0.037	0.002		0.045
Fatty acid metabolism	0.037		0.049	0.033
Nucleotide excision repair	0.001		0.008	0.005
Renal cell carcinoma		0.024	0.015	
Protein export		0.053	0.024	
Steroid biosynthesis			0.004	0.034
Fc epsilon RI signaling pathway		0.006		0.046
Jak-STAT signaling pathway		0.003		0.048
MAPK signaling pathway		0.009		0.023

a KEGG pathways that are significant at ≥ 2 doses.

b FDR-adjusted *p*-value (Benjamini and Hochberg 1995) < 0.005 in overall analysis. Details of all KEGG pathways are available from Kyoto Encyclopedia of Genes and Genomes (2000).

**Table 4:** Potential biomarkers of benzene exposure based on gene expression ratios relative to unexposed controls.

			Benzene exposure category							
			Very low (*n* = 29)		Low (*n* = 30)		High (*n* = 11)		Very high (*n* = 13)	
Probe ID	Symbol	Definition	Ratio	*p-*Value^a^	Ratio	*p-*Value^a^	Ratio	*p-*Value^a^	Ratio	*p-*Value^a^
5090327	*SERPINB2*^b^	serpin peptidase inhibitor, clade B, member 2	2.47	0.002	5.19	0.000	3.03	0.005	3.39	0.001
2370524	*TNFAIP6*	tumor necrosis factor, alpha-induced protein 6	2.26	0.000	2.94	0.000	1.72	0.030	2.13	0.000
6590338	*IL1A*^b^	interleukin 1, alpha	2.00	0.001	3.03	0.000	2.36	0.000	2.53	0.000
1260746	*KCNJ2*	potassium inwardly-rectifying channel, subfamily J	1.97	0.000	2.54	0.000	2.09	0.000	1.56	0.012
2230131	*PTX3*^b^	pentraxin-related gene, rapidly induced by IL-1 beta	1.80	0.000	2.30	0.000	1.62	0.003	1.81	0.000
5860333	*F3*	coagulation factor III (thromboplastin, tissue factor)	1.73	0.003	2.83	0.000	1.78	0.034	2.41	0.001
1410189	*CD44*^b^	CD44 antigen (Indian blood group)	1.64	0.000	1.76	0.000	1.64	0.005	1.78	0.000
2470100	*CCL20*	chemokine (C-C motif) ligand 20	1.63	0.005	2.30	0.000	1.59	0.041	2.11	0.000
4880717	*ACSL1*	acyl-CoA synthetase long-chain family member 1	1.63	0.001	1.79	0.000	1.59	0.010	1.68	0.002
1470682	*PTGS2*^b^	prostaglandin-endoperoxide synthase 2	1.60	0.000	1.98	0.000	1.68	0.003	1.75	0.000
1770152	*CLEC5A*	C-type lectin domain family 5, member A	1.57	0.009	2.26	0.000	1.78	0.014	2.26	0.000
4060674	*IL1RN*	interleukin 1 receptor antagonist	1.55	0.003	2.26	0.000	1.54	0.020	1.61	0.004
7320646	*PRG2*	proteoglycan 2, bone marrow	1.37	0.011	1.83	0.000	1.5	0.007	1.69	0.000
650709	*SLC2A6*	solute carrier family 2, member 6	1.36	0.005	1.72	0.000	1.5	0.000	1.60	0.000
2900286	*GPR132*	G protein-coupled receptor 132	1.34	0.047	1.87	0.000	1.6	0.003	1.80	0.000
3710379	*PLAUR*	plasminogen activator, urokinase receptor	1.29	0.035	1.80	0.000	1.6	0.002	1.58	0.001

Genes shown are up- or down-regulated ≥ 1.5-fold relative to unexposed controls at three or four doses.

a FDR-adjusted *p*-value (Benjamini and Hochberg 1995).

b Genes that have *p*-values ≤ 0.005 at all four doses.
